# Progression in the Oxidation Stability of MXenes

**DOI:** 10.1007/s40820-023-01069-7

**Published:** 2023-04-18

**Authors:** Razium A. Soomro, Peng Zhang, Baomin Fan, Yi Wei, Bin Xu

**Affiliations:** 1 State Key Laboratory of Organic-Inorganic Composites, Beijing Key Laboratory of Electrochemical Process and Technology for Materials, Beijing University of Chemical Technology, Beijing, 100029 People’s Republic of China; 2 College of Chemical and Materials Engineering, Beijing Technology and Business University, Beijing, 100048 People’s Republic of China

**Keywords:** MXenes, Oxidation stability, Antioxidation strategies, Shelf-life of MXenes

## Abstract

MXenes are under the spotlight due to their versatile physicochemical characteristics. Since their discovery in 2011, significant advancements have been achieved in their synthesis and application sectors. However, the spontaneous oxidation of MXenes, which is critical to its processing and product lifespan, has gotten less attention due to its chemical complexity and poorly understood oxidation mechanism. This perspective focuses on the oxidation stability of MXenes and addresses the most recent advancements in understanding and the possible countermeasures to limit the spontaneous oxidation of MXenes. A section is dedicated to the presently accessible methods for monitoring oxidation, with a discussion on the debatable oxidation mechanism and coherently operating factors that contribute to the complexity of MXenes oxidation. The current potential solutions for mitigating MXenes oxidation and the existing challenges are also discussed with prospects to prolong MXene’s shelf-life storage and expand their application scope.

**Graphical Abstract:**

## Highlights

- The progression of MXene's oxidation stability, the techniques available to monitor the phenomenon as well as the variables that contribute to its oxidation rate are discussed.
- Comprehensive aspects of the oxidation process in various storage settings and the debated oxidation mechanism along with the most effective antioxidation strategies are addressed in conjunction with current challenges to the air stability of MXenes.

## Graphical abstract

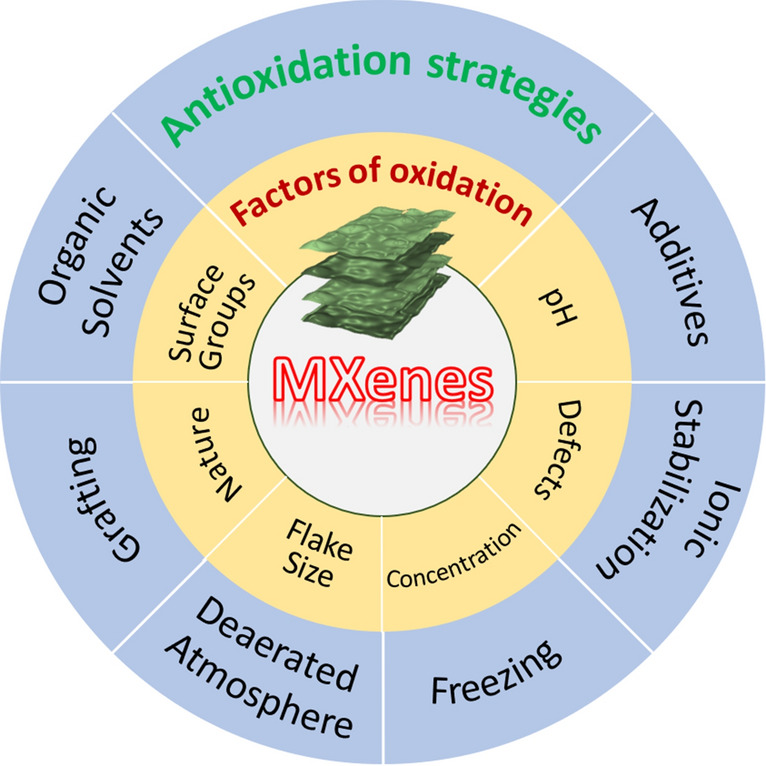

## Introduction

MXenes, a family of 2D materials composed of carbides, nitrides, and carbonitrides, are in the spotlight on account of their remarkable properties [1–3]. Since their discovery in 2011, many chemical configurations have been identified, with over 100 different stoichiometric compositions theoretically predicted and over 40 experimentally synthesized [4, 5]. MXenes are represented by a general formula M_*n*+1_X_*n*_T_*x*_, where M stands for early transition metals, X is carbon or/and nitrogen atoms, *n* = 1–4 and T_*x*_ represents associated surface terminations (–F, –O, –OH, etc.) [6–9]. After completing a 10-year milestone, MXenes are progressively recognized in many science and technology areas due to their high conductivity, large surface aspect ratio, high mechanical strength, diverse active surface terminations, and excellent hydrophilicity [5, 10–12]. Though the application domain of MXenes is constantly expanding, their low chemical stability, particularly in aqueous dispersion form, is a key barrier to their shelf-life storage and product lifespan [13].

Currently, storing aqueous MXenes without oxygen, at low temperatures, and without light radiation is suggested to extend their shelf-life. Although successful to some extent, storage condition modification complicates MXenes processing and the operating settings of MXene-based devices. Many alternative strategies have been explored to improve the oxidation stability of MXenes, ranging from reducing their terminal groups to using antioxidants and chemical grafting. However, finding a method to halt the spontaneous aqueous oxidation of MXenes remains challenging. In the case of Ti_3_C_2_T_*x*_, one of the most commonly explored MXenes, this oxidation is reported to occur due to water and dissolved oxygen’s interaction with the flake edges and defects, resulting in TiO_2_ nucleation, which then grows and spreads throughout the surface [6, 14]. MXenes oxidation stability may be improved by developing innovative structural modulation and protection routes. The major underlying challenges, in this regard, are primarily due to the lack of complete atomic-scale understanding of MXenes oxidation, which has resulted in a debatable interpretation of its mechanisms, as well as the infancy of molecule-MXene interactional chemistry, which is critical for developing effective stabilizing strategies.

As MXenes are promising next-generation materials [15, 16], it is necessary to address this bottleneck issue of oxidation stability before MXenes could lead the way for many diverse applications. Herein, we provide a perspective on the oxidation stability of MXenes in their decade of progression. An overview of presently used methods for detecting and monitoring/tracking oxidation is followed by a special section on the debatable oxidation mechanism of MXenes and the most current strategies proven effective in prolonging their aqueous shelf-life storage. Finally, endeavors are directed at existing MXene oxidation stability challenges and prospects to establish a clear path towards developing new efficient methods to stabilize MXenes and broaden their application spectrum.

## Monitoring the Oxidation of MXenes

A strong correlation between MXenes’ fundamental characteristics and their behavior under various storage conditions is vital for their advancement toward practical purposes. To achieve this, it is imperative to evaluate MXenes structure, surface chemistry and compositional characteristics using reliable techniques or methods that are critical in assessing the origin and specific characteristics of MXenes under oxidation conditions. Among the prominent techniques, X-ray and microscopic methods, such as X-ray diffraction (XRD), scanning electron microscopy (SEM), transmission electron microscopy (TEM) coupled selected area electron diffraction (SAED), and X-ray photoelectron spectroscopy (XPS) along with spectroscopic techniques such as Raman and UV–Vis spectrophotometry could be employed to monitor the oxidation status of MXenes.

**Fig. 1:**
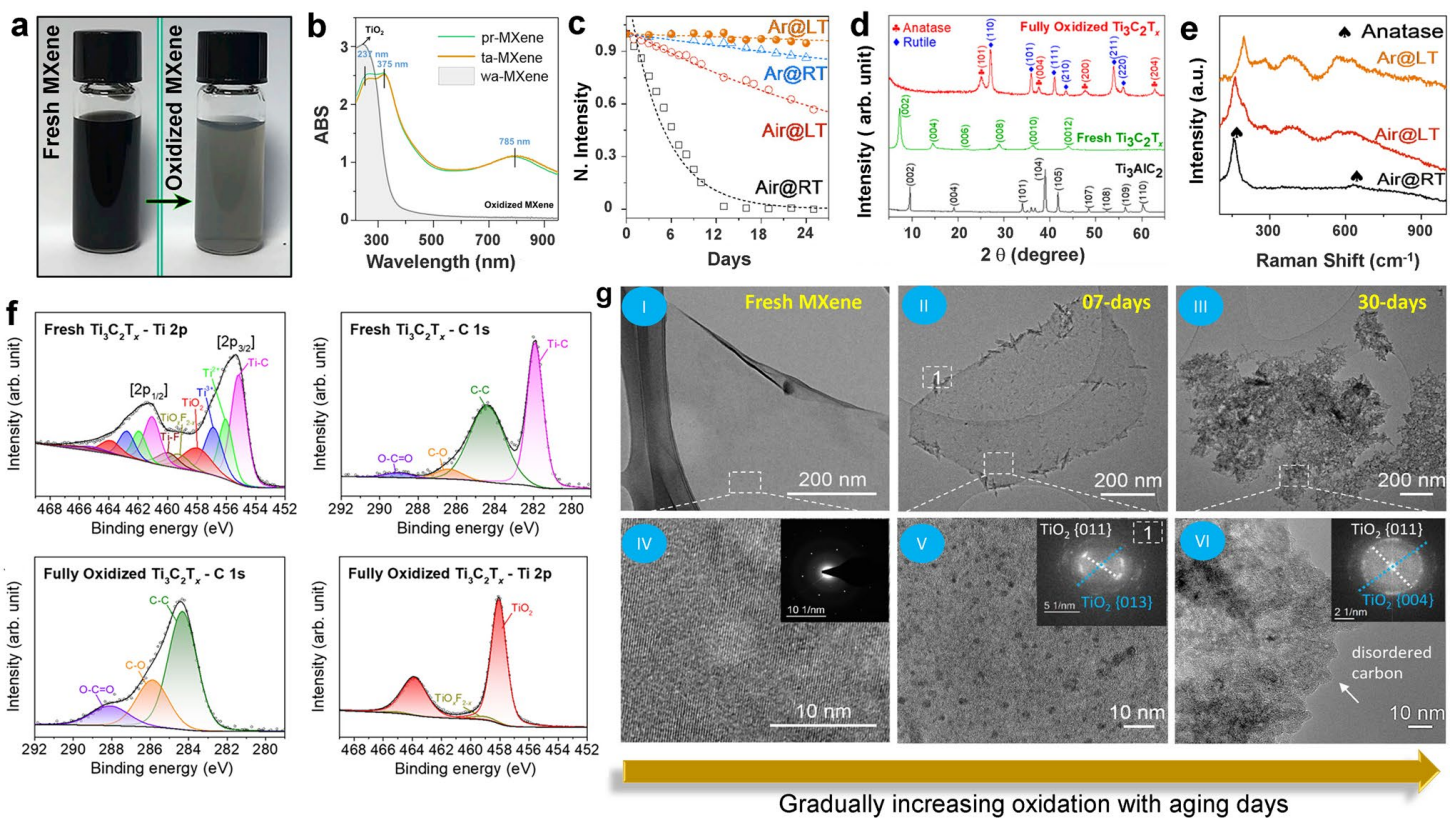
**a–b** Digital photograph depicting Ti_3_C_2_T_*x*_ color change during oxidation and corresponding UV–Vis absorption spectrum profile with typical abs peaks of Ti_3_C_2_T_*x*_ and its oxidized counterpart [19]. Copyright 2021, Elsevier. **c** Variation in normalized abs intensity of Ti_3_C_2_T_*x*_ with different colloidal environments and temperatures during 24 days of storage [18]. Copyright 2017, American Chemical Society. **d** XRD pattern of Ti_3_AlC_2_, Ti_3_C_2_T_*x*_ and its oxidized counterpart showing the presence of typical TiO_2_ (Anatase/rutile) at the expense of MXenes 002 peak [20]. Copyright 2021, American Chemical Society. **e** Raman spectrum depicting oxidative changes in Ti_3_C_2_T_*x*_ in different environments [18]. Copyright 2017, American Chemical Society. **f–g** Difference of XPS high-resolution Ti 2*P* and C1*s* for fresh and oxidized Ti_3_C_2_T_*x*_ and the TEM images of Ti_3_C_2_T_*x*_ during its 30 days of storage in aqueous media showing the gradual formation of TiO_2_ particles [20]. Copyright 2021, American Chemical Society

Since substantial research is devoted to comprehending the oxidation of Ti_3_C_2_T_*x*_-MXene, this discussion thoroughly focuses on monitoring its oxidation. The most common way to detect oxidation is to observe the change in Ti_3_C_2_T_*x*_ dispersion from dark green/black to hazy white with the naked eye (Fig. 1a). Though suitable for primary observation, color change in MXenes is subject to considerable variability and should thus be accompanied by further analysis of the Ti_3_C_2_T_*x*_ optical and compositional properties to accurately identify its degree of oxidation. One of the quickest and easiest ways to monitor the occurrence of oxidation in MXenes colloidal form is through UV–Vis spectroscopy [17]. This method, which relies on measuring the absorption of electromagnetic radiation in the ultraviolet and visible region, can reveal important information about structural and/or compositional variations, particularly for the low concentration of MXenes based on the changes in their specific optical band positions and intensities. In the case of Ti_3_C_2_T_*x*_ as the oxidation progresses, the edge-formed TiO_2_ particles grow and spread throughout the layered structure resulting in a proportional decline in the concentration of MXene-flakes and their size. This physical change could be verified by tracking the optical characteristics of Ti_3_C_2_T_*x*_-MXene dispersion using a simple UV–Vis spectrophotometer, where changes in its typical absorption peaks (∼235, ∼375, and ∼785 nm) could be used to determine the kinetics of its oxidation by using the following empirical equation:

1 $$ A = A_{{{\text{stable}}}} + A_{{{\text{unstable}}}} e^{ - t/\tau} $$

Here, *A*, *A*_stable_, and *A*_unstable_ are the abs of stable and unstable MXene, while “*t*” and “*τ*” represent time and time constant (days). As the oxidation progresses, the representative band at 785 nm decreases while the 237 nm band, representative of TiO_2_, gradually increases (Fig. 1b). Thus following the optical abs at 785 nm, it is possible to estimate the concentration of Ti_3_C_2_T_*x*_-MXene dispersion at any given time and condition (Fig. 1c). Hence, the oxidation status of Ti_3_C_2_T_*x*_ could be monitored against various factors such as dispersion environment (solvent and concentration), pH, and temperature. This tracking of MXenes oxidation via changes in its optical characteristics offers a viable route to establish a quantitative relationship between flake size and stability, and thus could be generalized for size selection of MXene flakes and assessment of their dispersion quality [18]. XRD is a powerful nondestructive tool for the crystallographic evaluation of MXenes and thus could be relied upon to monitor the progress of the MAX phase etching, layer intercalation and oxidation in their film/powder form. The bulk compositional status could be evaluated by XRD, where the typical MXene peaks and their intensities diminish proportionately as oxidation increases, while characteristics peaks of TiO_2_ (anatase/rutile) emerge, whose intensities are comparable to their relative content in the final oxidized product (Fig. 1d). While XRD provides sensitive crystal and *d*-spacing information of MXenes, it is unsuitable for evaluating minimum surface changes, such as early oxidation phases near the edge of MXene nanosheets in aqueous MXenes dispersion. In this case, Raman spectroscopy, which is sensitive to lattice vibrations, could generate a molecular fingerprint of MXenes providing necessary information regarding changes in bonding and the presence of trace transition metal oxides, enabling sophisticated, rapid and sensitive detection of MXenes oxidation. In the case of Ti_3_C_2_T_*x*_ film, changes in the typical Raman peak of Ti_3_C_2_T_*x*_ near 205 cm^−1^ with emerging TiO_2_ vibration frequencies near 150 cm^−1^ could also be tracked to monitor the oxidation of MXene (Fig. 1e). XPS analysis is often used to determine the chemical stability of the bonds inside MXenes. This surface-based approach offers multiple measurement benefits, including the assessment of MXenes elemental status, surface components (functionality), and the trace oxide product generated due to the surface/edge oxidation of MXenes. Thus, XPS is the chosen technique for quantifying MXene surface oxidation.

Regarding Ti_3_C_2_T_*x*_, the diminishing bond-energy intensities of Ti-C and other Ti-associated energies in the deconvoluted Ti 2*p* spectra of MXenes could be used to trace the steadily growing TiO_2_. This, coupled with the increase in the C–C energy band after the decline of Ti-C binding energy in the matching MXene C 1*s* profile, might offer an accurate estimate of MXene’s degree of oxidation (Fig. 1f). In addition to corroborating the effective etching of the MAX phase, microscopic methods like SEM and TEM may be used to evaluate morphological characteristics such as flake shape and size in addition to visualizing the early development of transition-oxides in MXenes (oxidation). TEM, combined with selected area electron diffraction (SAED), may disclose information regarding the atomic arrangement, interlayer spacing, and surface defects of MXenes nanosheets, which are crucial characteristics for evaluating the compositional and oxidative stability of MXenes. The advancement of oxidation enhances the growth of TiO_2_ on MXene, resulting in noticeable MXene-morphological changes with increasing particle (TiO_2_) density. Here, microscopic techniques such as TEM could assist in determining if MXene has undergone mild, partial, or total oxidation by assessing the TiO_2_ particle distribution, size, and location (edge/or surface), whereas the corresponding SEAD pattern, could assist in determining the lattice parameters, and crystal structure of the oxidized product (TiO_2_) (Fig. 1g).

In addition to these methods, other indirect routes, such as a change in the conductivity of MXenes due to the resistive nature of TiO_2_, have also been investigated to measure the oxidation state. However, since MXene conductivity is also sensitive to film thickness and MXene-layer alignment, a direct correlation of conductivity with oxidation may result in incorrect conclusions. The analytical techniques mentioned above might be collectively employed to understand and quantify MXenes oxidation level both in dispersion form or solid (film) and could assist in exploring new strategies capable of enhancing MXenes shelf-life storage.

## Influencing Factors for Oxidation Stability of MXenes

The oxidation stability is crucial for MXenes widespread applicability as they tend to transform into their oxide derivate in an aqueous environment. This transformation degrades their unique 2D structural features and results in the loss of physicochemical properties. To date, several efforts have been directed toward understanding and extending the oxidation stability of titanium-based MXenes, notably by investigating their storage conditions, such as dispersion environment, pH, and temperature.

The first exfoliated MXene, i.e., Ti_3_C_2_T_*x*_*,* was thought to gradually deteriorate into TiO_2_ due to its reaction with dissolved oxygen in the aqueous system. Huang et al. [21] questioned the fundamental of the oxidation mechanism, which had significant implications for MXenes shelf-life storage. During the 13 days of the oxidation stability study, Ti_3_C_2_T_*x*_ remained reasonably stable in isopropanol/air. The paper reasoned that oxidation should occur faster in isopropanol as it has a higher mole fraction of soluble oxygen (7.82 × 10^–4^). However, UV–Vis and Raman spectroscopy observations revealed contradicting results, and thus the role of water was changed from being a solvent to more like a reagent owing to Ti_3_C_2_T_*x*_ deterioration even in an inert atmosphere. Zhang et al. [18] later proposed that this oxidation initiated from the edges of the Ti_3_C_2_T_*x*_ flakes following single exponential degradation kinetics. The kinetics of MXenes degradation was evaluated in various environments (air and argon) to propose an efficient route to improve their stability in a natural aqueous state. The change in the optical characteristic (UV–vis abs) of Ti_3_C_2_T_*x*_ at a fixed concentration under different environments (air and argon) led to the conclusion that storing MXenes under an inert atmosphere and at lower temperatures could suppress the oxidation. The correlation established a relationship between Ti_3_C_2_T_*x*_ flake size and degradation rate constant, enabling accurate flake size estimation using a simple UV–Vis spectrophotometer. Since dissolved oxygen in an aqueous Ti_3_C_2_T_*x*_ dispersion is more reactive at the edges of the flakes, smaller flakes oxidize faster than bigger ones. Thus, increasing the lateral flake size and altering the colloidal dispersive environment from room temperature to refrigerated and deaerated setting was proposed as a viable route to increase the shelf-storage period of aqueous Ti_3_C_2_T_*x*_.

**Fig. 2:**
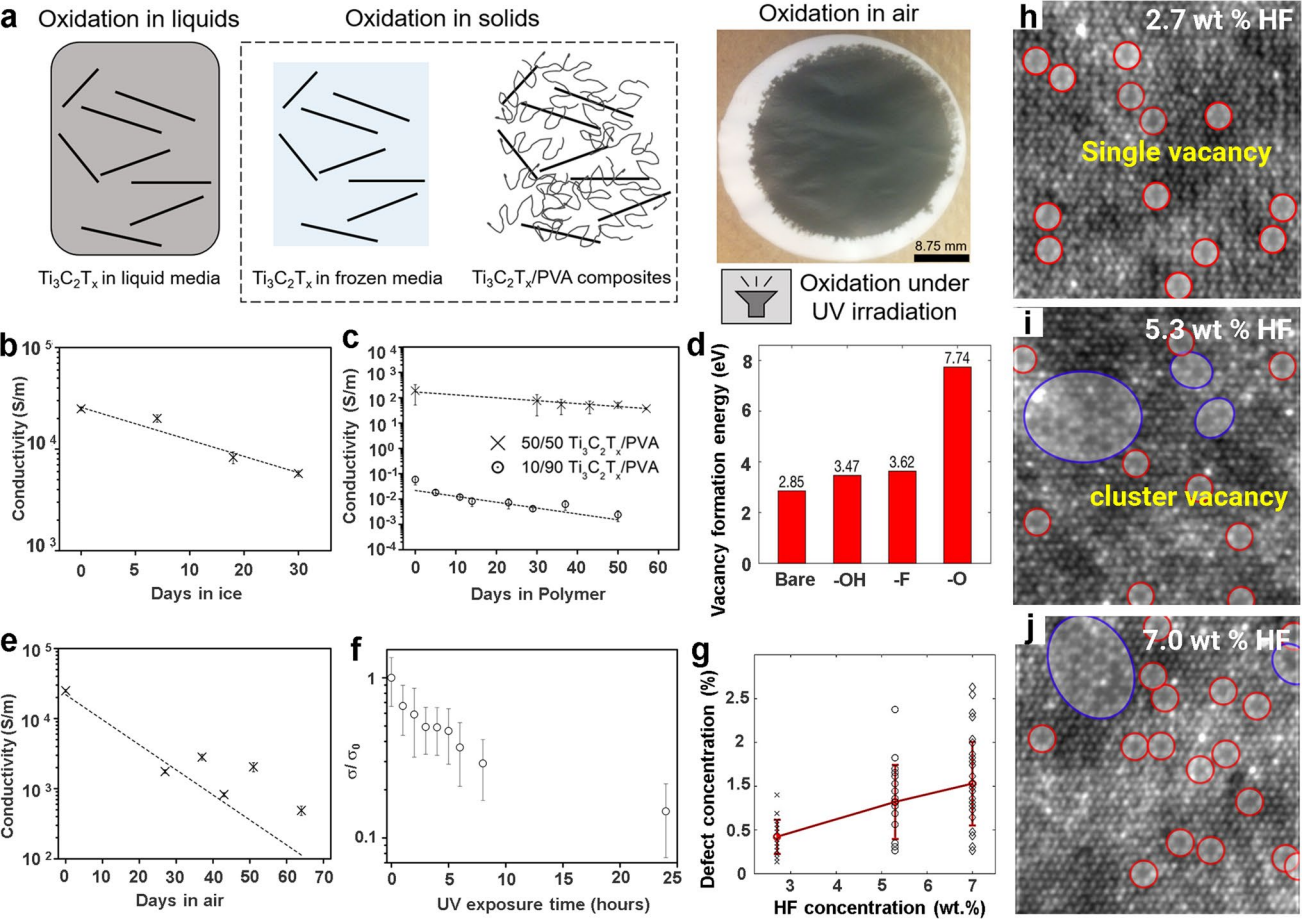
**a** Schematic illustration representing oxidation of Ti_3_C_2_T_*x*_ in liquids, solids and air medium, **b-c** change in conductivity of Ti_3_C_2_T_*x*_ films produced from the dispersion stored in ice, polymer and air respectively [22]. Copyright 2019, Springer Nature. **d** Variation of vacancy formation energy for Ti_3_C_2_T_*x*_ with different surface functionalities, **e–f** change in resistance of Ti_3_C_2_T_*x*_ films in air and films produced from aqueous media during UV-exposure for 25 h and, **g** defect concentration variation and corresponding, **h-j** HR-TEM images showing single and cluster vacancies formation with increasing HF concentration [23]. Copyright 2016, American Chemical Society

The influence of storage environment and light on Ti_3_C_2_T_*x*_ oxidation was further explored against its conductivity. Habib et al. [22] studied the storage of Ti_3_C_2_T_*x*_ in different mediums, including air, liquid (water, acetone, and acetonitrile) and solid form (ice and polymer) (Fig. 2a). Since oxidation can reduce MXene’s intrinsic properties, conductivity loss was used as a more dependable indicator than the colloidal color change. Unlike fresh vacuum-dried aqueous MXene nanosheets (Fig. 2e), films formed from acetone and acetonitrile dispersions retained their conductivity even after 21 days in ambient air, indicating a delayed oxidation process. Films prepared from colloidal dispersion maintained at 0 °C (ice) (Fig. 2b) displayed conductivity change similar to that of fresh MXene films, whereas polymer (PVA) driven films had slower conductivity loss than the water equivalents (Fig. 2c). A similar pattern was found for Ti_3_C_2_T_*x*_ kept in the dark and under UV irradiation, with the latter losing 87% of its original conductivity within 24 h and the former taking 27 days (Fig. 2f). This rapid conductivity loss under illumination was attributed to the accelerated oxidation of Ti_3_C_2_T_*x*_ due to heat generation. In addition, under illumination, TiO_2_ photo excites and produces O⋅ and OH⋅ radicals which further accelerates the oxidation of MXenes. While managing the storage settings, i.e., light, atmosphere, and temperature, may assist in extending the shelf-life of Ti_3_C_2_T_*x*_, total oxidation inhibition was almost impossible in all conditions.

Since several factors contribute to this oxidation phenomenon, a significant disparity is observed mainly for the storage lifespan of Ti_3_C_2_T_*x*_. For example, aqueous Ti_3_C_2_T_*x*_ at low concentration and smaller flake size oxidize faster owing to their high dispersibility with much-exposed surfaces and edges to dissolved oxygen and water molecules. In contrast, the concentrated dispersion has relatively less exposed surfaces and edges owing to steric shielding between its flakes. Furthermore, research also supports modest oxidation mitigation in an aqueous state when MXenes are stored undisturbed, compared to its agitated equivalent, which has a low steric shielding effect [24]. Because several factors contribute to MXenes oxidation in an aqueous environment, a complete understanding of the MXenes oxidation process remains elusive.

The susceptibility of MXene flakes and their edge defects to water or oxygen is another perspective to view and understand MXenes’ oxidation. Taking the example of well-studied MXene, i.e., Ti_3_C_2_T_*x*_, which is commonly produced using a conventional acid etching route involving strong acid, such as HF, to produce accordion-like MXenes, which could be further delaminated into mono or few-layer MXenes via sonication or intercalating foreign species (i.e., DMSO). In the case of Ti_3_C_2_T_*x*_, the direct use of harsh acids such as HF is the main route to form defects. As the etching progresses, some of the existing defects of the precursor MAX phase are inherited into their layered counterpart [25]. Thus the quality of the MAX phase is critical in controlling the defect concentration in the produced MXenes. In addition, cationic vacancies arise due to the direct interaction of MXene layers with acid during the etching of the "A" layers from the MAX phase [26]. These Ti-vacancies occur at the top and bottom layers, which are more acid-exposed than the core (bulk) layers, with Ti layers next to the C layers, implying that vacancy formation, particularly in a few layers of Ti_3_C_2_T_*x*_, is directly related to HF concentration (Fig. 2g-j). Moreover, the lower vacancy formation energy (2.842 eV) for the outer layers compared to the inner layers (6.485 eV) revealed that the former layers are more susceptible to a higher defect formation when exposed to a stronger etchant. Therefore, the oxidation susceptibility of MXenes in the aqueous dispersion is directly related to the defect generation, which is associated with synthesis condition (-HF etching or MILD), MAX phase purity, and etching duration. For example, oxidation will occur more rapidly in smaller flakes with more defects than in larger flakes with few defects. The oxidation susceptibility of MXenes could also be correlated to the number of the formed MXene-layer (n in M_*n*+1_X_*n*_T_*x*_), particularly for MXenes with similar chemistries [27]. As the number of the MXene-layers is related to the stiffness of M_*n*+1_C_*n*_ layers, the greater number of layers would exert more mechanical stress and limit the intercalation of ions during their etching process [28]. For example, using 10% HF, the Ti_2_CT_*x*_ (*n* = 1) requires 10 h of etching from the corresponding MAX phase, whereas the Ti_3_C_2_T_*x*_ (*n* = 2) needs 18 to 24 h under similar conditions [29]. Consequently, MXenes with multiple layers of M and X (higher *n*) would be more thermodynamic stable [30] and be less susceptible to oxidation owing to their much less exposed surfaces with fewer cationic vacancies and defects than the monolayered counterpart.

The type of surface functional terminations has also been connected to vacancy formation in MXenes. Here Ti_3_C_2_T_*x*_ with –F and –OH terminations exhibited lower vacancy formation energy than –O terminations (Fig. 2d). Since the etchant composition directly determines the nature of surface functionalities in Ti_3_C_2_T_*x*_, defect concentration and flake size could be regulated by choosing an appropriate synthesis route. For example, direct HF etching produces –F and –OH dominated MXenes compared to the in situ HF method, where –O terminations are abundant. Therefore, milder synthesis techniques with larger flake sizes ought to provide surface characteristics with fewer defects and, thus, higher oxidation stability.

The thermo-catalytic potential of MXenes has led to research on their high-temperature stability. According to the temperature and whether MXenes remain in the final product, the oxidation of MXenes is classified as partly or totally oxidized at high temperatures. However, the use of ex situ techniques such as XRD, Raman, and XPS is limited to providing only the post-oxidative information of the MXenes. Ghassemi et al. [31] initially observed the in situ formation of TiO_2_ using TEM by oxidizing Ti_3_C_2_T_*x*_ in the air. The flash oxidation of MXene (950 °C) revealed the formation of anatase particles, while rutile sheets formation was observed for slow oxidation (450 °C using 0.1 °C s^−1^). This initial study enabled the development of MXene’s original temperature stability profile and indicated that changing the temperature rate and exposure period could control the rate of oxidation and, thus, compositional stability. Since then, the oxidation of MXenes has been examined at different temperatures and in various environments, including air, argon, and ammonia. Li et al. [32] observed that Ti_3_C_2_T_*x*_ with -F and -OH termination remained stable till 800 °C in argon and 200 °C in oxygen. While in the case of ammonia, the oxidation process was comparable to that of an inert environment. However, the C atoms were replaced with N atoms transforming MXene into *N*-doped Ti_3_C_2_T_*x*_ [33]. Rakhi et al. [34] further studied the oxidation of Ti_2_CT_*x*_ under Ar, N_2_ and N_2_/H_2_ mixture with implications to MXenes electrochemical properties. The study confirmed the formation of TiO_2_ and graphitic carbon when MXenes were annealed at 226.85 °C for 2 h in the air while maintaining their structural integrity in N_2_, N_2_/H_2_ and Ar atmospheres.

Wang et al. [35] studied the oxidation behaviour of Ti_3_C_2_T_*x*_, V_2_CT_*x*_, Nb_2_CT_*x*_ and Mo_2_CT_*x*_ in different atmospheres and temperatures. In argon, Ti_3_C_2_T_*x*_ was thermally stable between 800 and 1,200 °C with some edge-based TiO_2_ formation. Though the preservation of Ti_3_C_2_T_*x*_ could be achieved at 1,200 °C, a phase transformation to cubic TiC_*x*_ was observed [36]. More recently, Seredych et al. [37] studied the change in MXenes compositional characteristics with temperature using a combination of TGA and mass spectrometry. The TGA profiling of Ti_3_C_2_T_*x*_, Nb_2_CT_*x*_, and Mo_2_CT_*x*_ at different temperatures and the analysis of their post-oxidization products enabled insights into MXenes degradation. Accordingly, for all MXenes, the temperature change resulted in their mass loss, and the process was classified into four main stages. For example, Ti_3_C_2_T_*x*_ showed the highest thermal stability with the first gradual weight loss region observed till 370 °C, which was attributed to the release of layer-entrapped water molecules generating strong mass spectroscopy signals of H_2_O, –OH, and = O. As the temperature rises to 530 °C, the slow dissociation of function groups occurs, generating H_2_ and O_2_ signals from the combination of –OH surface groups and the molecular hydrogen within MXene layers. The degradation region was defined above 740 °C in association with an intense signal of CO, indicating the transformation of MXenes into cubic TiC form, which upon further temperature increment, releases fluorine in the form of HF and AlF_3_.

Interestingly, the change in synthesis conditions, such as the concentration of HF from 5 to 30%, did not affect the degradation temperature (> 740 °C). However, a significant influence on the surface chemistry was evident as higher HF concentration resulted in the generation of higher fluorine-related gaseous products. In comparison with Ti_3_C_2_T_*x*_, the Nb_2_CT_*x*_, and Mo_2_CT_*x*_ also followed a similar degradation trend, but their degradation temperatures were much lower, and mass spectrometry verified the presence of abundant –O groups but no -F termination. The study demonstrated that the etchant composition, i.e., a mixture of acids such as HF/HCl or HF/H_2_SO_4_, had a significant influence on the surface chemistry of Ti_3_C_2_T_*x*_. Consequently, a distinct deterioration profile might be envisioned for MXenes derived from the same MAX phase by utilizing different etchants.

MXenes oxidation is a complicated phenomenon in which several factors, such as pH, size of MXene-sheets, concentration, environment, and MXenes nature, coherently influence the rate of its oxidation. Therefore, proper control of the experimental conditions is crucial to retard the oxidation of MXenes in the dispersion state and extend its shelf-life stability.

## Debate on the Oxidation Mechanism of MXenes

It has been recognized that water and dissolved oxygen are the main culprits to MXene oxidation in aqueous dispersion, whereas the rate of its degradation depends on various factors, including flake size, defects, morphology (accordion/few-layer), MAX phase quality, and storage conditions (dark or light). Although efforts have been made to understand more about the oxidation kinetics of MXenes, especially Ti_3_C_2_T_*x*_, under different storage settings and environmental conditions, the actual oxidation mechanism still remains under debate. Some studies stress the role of dissolved oxygen, whereas others identify water as the primary contributor. Regardless, the fundamental understanding of how these culprits interact with MXenes in relation to adequate experimental data remains unclear.

**Fig. 3:**
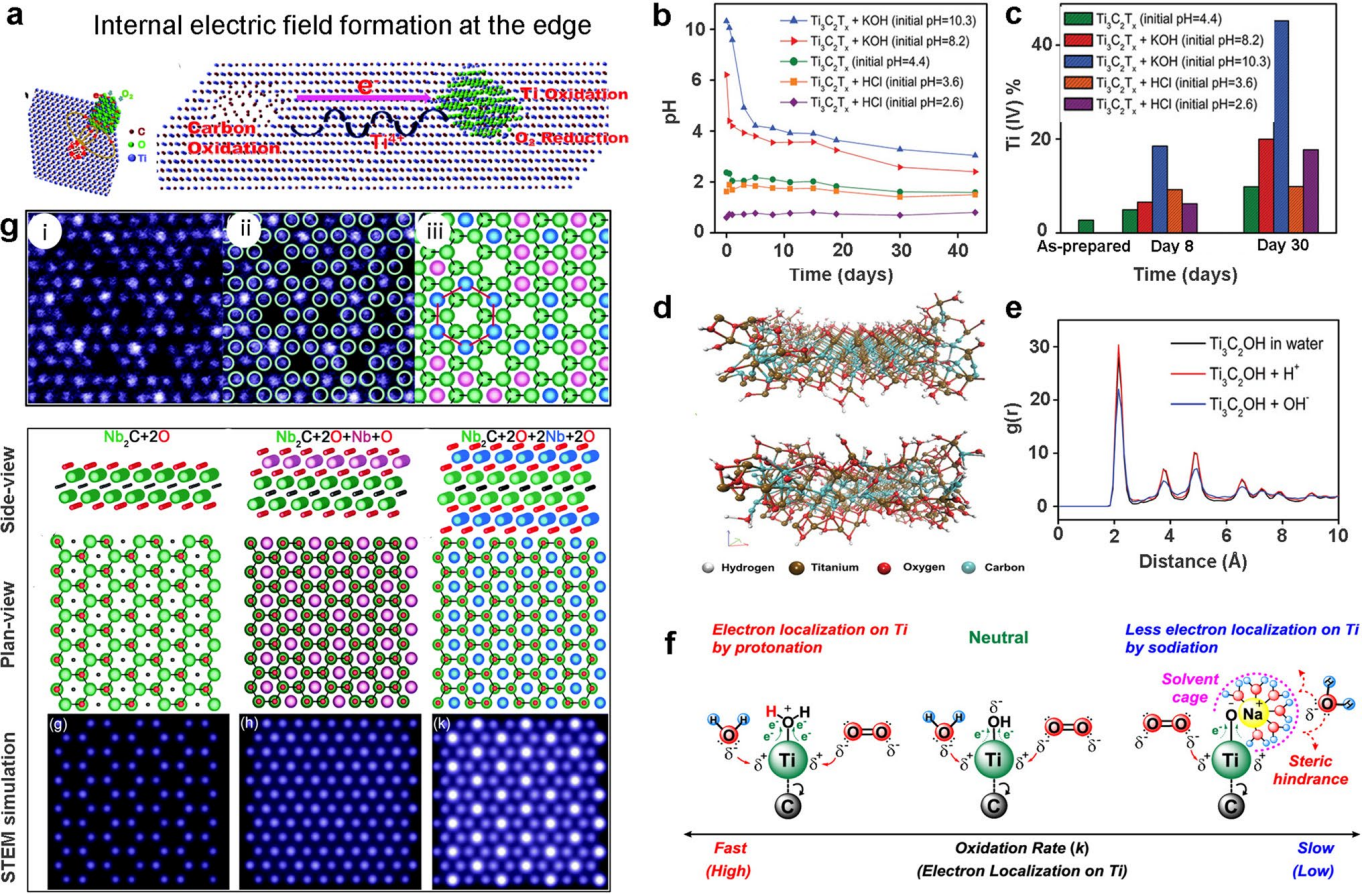
**a** Illustration of internal electric field formation in Ti_3_C_2_T_*x*_ [38]. Copyright 2019, Royal Society of Chemistry. **b** Variation of colloidal MXene’s pH against time and **c** corresponding atomic percentage alteration for Ti(IV) component. **d** Molecular configuration of Ti_3_C_2_T_*x*_ based on 25 ps ReaxFF analysis with overhead configuration for MXene acidic and lower for the alkaline environment [24]. Copyright 2021, Wiley–VCH. **e** Radial distribution function (rdf) peaks for Ti-C bonds in acidic, alkaline, and water-based MXene systems. **f** A general illustration for the oxidation of Ti_3_C_2_T_*x*_ and Ti_2_CT_*x*_ MXene nanosheet against acidic and alkaline pH [20]. Copyright 2021, American Chemical Society **g** Atomically-resolved STEM image recorded for Nb_2_CT_x_ MXene with (i) overlaid M_2_X model structure and (ii) corresponding crystal structure sketch with a representative honeycomb core highlighted with a red line; along with side and plane-view crystal structure projections and STEM simulations for Nb_2_C model structure with O-termination [26]. Copyright 2018, Royal Society of Chemistry

Some studies have attempted to clarify the oxidation process in MXenes. Among the first, Xia et al. [38] offered a much deeper understanding of Ti_3_C_2_T_*x*_ oxidation and charted the oxidation mechanism based on the susceptibility of Ti vacancies to dissolved oxygen. Taking advantage of STEM and electron energy loss spectroscopy (EELS) analysis, the formation of TiO_2_ at the edge defects was studied at normal room temperature conditions. Accordingly, as the Ti-vacancies form (Fig. 3a), electrons tend to flow toward the defects, following the generation of an internal electric field that promotes the nucleation of carbon clusters. Since the vacancies are positively charged, the nearby carbon atoms (C_4_^−^) tend to oxidize (lose electrons), accumulating electron holes around the defect sites and facilitating further oxidation. Since electron holes are positive and the defects are negative, an internal electric field pulls the Ti-atom toward the negative side, enabling the dissolved O_2_ (in the form of O_2_^−^) to interact with the MXene lattice plane and generate TiO_2_ while carbon oxidizes toward the opposing end of the field (Fig. 3a). Interestingly, the study proposed that the internal field near Ti-vacancy causes Ti-atom diffusion, at the same time, the electron–hole transportation inside the layers was responsible for the formation and spread of TiO_2_ particles. Moreover, the formed TiO_2_ particles preferrable grow along the (101) lattice planes owing to its low-surface energy (0.4444 J m^−2^) perpendicular to the basal plane (0001) of the Ti_3_C_2_T_*x*_ flake. In another effort, Zhao et al. [24] monitored the oxidation of Ti_3_C_2_T_*x*_ and Ti_2_CT_*x*_ with the change in pH and proposed an oxidation mechanism from the perspective of MXenes pH change. Interestingly, when placed in highly alkaline conditions for nearly 30 days, the change in MXenes’ initial pH is rapid, whereas, in the acidic medium, the change follows a gradual declining trend. Figure 3b compares this pattern to the reference dispersion of MXenes (initial pH 4.4), which follows a declining trend with time. The pH change, when corroborated with XPS investigation after 30 days of storage, indicated a considerable rise in the atomic percentage of Ti (IV) for alkaline compared to the acidic medium (Fig. 3c). The conductivity also followed a similar trend, dropping from (1.6 ± 0.78) × 10^5^ to (4.1 ± 0.1) × 10^4^ S m^−1^ for pH 10 dispersion in 18 days and eventually fading out in 30 days. Interestingly, Huang et al. [21] previously postulated that the pH decline (acidic nature) of the Ti_3_C_2_T_*x*_ in an aqueous setting is associated with forming the carbonic acid derivatives produced via MXene degradation. Though no experimental evidence was provided, their hypothesis was based on the transformation of generated carbon into CO_2_ and its dissolution in water, partially forming carbonic acid or derivatives as described by Eqs. (2) and (3):

2 $$ {\text{2Ti}}_{{3}} {\text{C}}_{{2}} {\text{O}}_{{2}} + {\text{11H}}_{{2}} {\text{O}} = {\text{6TiO}}_{{2}} + {\text{CO}} + {\text{CO}}_{{2}} + {\text{2CH}}_{{4}} + {\text{7H}}_{{2}} $$

3 $$ {\text{2Ti}}_{{3}} {\text{C}}_{{2}} \left( {{\text{OH}}} \right)_{{2}} + {\text{11H}}_{{2}} {\text{O }} = {\text{ 6TiO}}_{{2}} + {\text{CO}} + {\text{CO}}_{{2}} + {\text{2CH}}_{{4}} + {\text{9H}}_{{2}} $$

4 $$ {\text{Ti}}_{n + 1} {\text{C}}_{n} {\text{OH}} + {\text{OH}}^{ - } = {\text{ Ti}}_{n + 1} {\text{C}}_{n} {\text{O}}^{ - } + {\text{H}}_{{2}} {\text{O}} $$

Zhao et al. [24] proposed a mechanism speculating that in alkaline conditions, the hydroxyl anions react with the protons of MXenes -OH terminal groups, resulting in their deprotonation and forming less stable –O^−^ groups which make the surface more vulnerable to oxidation (Eq. (4)). In addition, the hydroxyl species could react with positively charged MXene edges [39], causing its degradation and TiO_2_ formation. These two hypotheses were supported by ReaxFF simulation performed for single-layer Ti_3_C_2_T_*x*_ in 3 hypothetical systems: water, hydroxyl ion in water, and protons in water. Here, the radial distribution function (RDF) peak for the T–C bond of Ti_3_C_2_T_*x*_ in an acidic medium was similar to that of the fresh Ti_3_C_2_T_*x*_ (Fig. 3d-e), indicating stable MXenes in acidic media owing to the formation of H_3_O^+^ which retards the oxidation compared to its alkaline counterpart. The study emphasized the critical role of OH^−^ radicals in oxidation. Thus, given a small MXene-flake with abundant -OH termination, edge-based interaction with OH^−^ radical might be faster than with water molecules, resulting in MXenes’ rapid deterioration. In contrast, excessively strong acidic conditions may result in more surface defects that function as reactive sites for oxidation; therefore, pH balance is vital for improving MXene storage life. A more detailed insight into the oxidation mechanism of Ti_3_C_2_T_*x*_ in correlation to its pH change was established by Doo et al. [20] based on the analysis of the gaseous species as the oxidation progresses. The GC-TOF–MS analysis of gaseous species at various pH and temperatures confirmed the generation of CH_4_, CO, and CO_2_ as main products during the oxidation of aqueous Ti_3_C_2_T_*x*_ set at pH 5.4 and 20 °C. Accordingly, these gaseous species were responsible for the pH decline as the oxidation proceeds following the protonation of hydroxyl functionalities on Ti_3_C_2_T_*x*_, reinforcing the electron localization toward the O atom of Ti–O–H bonds, as shown in Fig. 3f. The formed electron-deficient Ti atoms thus become more prone to be attacked from O_2_ and water molecules. On the other hand, hydroxyl groups would be deprotonated under alkaline conditions after developing Ti–O–Na^+^ intermediates, which are less electrophilic and may change into a hydrodynamic cage that might resist MXenes’ interaction with H_2_O and O_2_ due to the generation of steric hindrance, slowing the rate of oxidation in comparison to that observed in an acidic environment.

An atomic-level insight into the oxidation mechanism was proposed by Palisaitis et al. [26] where atomically resolved STEM and EELS were used to study the surface characteristics of Nb_2_CT_*x*_ MXene during its oxidation under storage conditions. After 5 days in ambient air, the STEM picture of Nb_2_CT_*x*_ showed a honeycomb-like atomic arrangement with growing structural disorders, as the corresponding FFT pattern suggested. Here confirmation of abundant oxygen element in samples cured for 5 days (-O/-OH termination) via EELS analysis further suggested a gradual deterioration of Nb_2_CT_*x*_ in ambient air. To interpret this oxidation process at an atomic level, a standard M_2_X model structure was projected along the c-axis and superimposed on the STEM image of Nb_2_CT_*x*_ by overlaying the honeycomb model cores above the image holes, as shown in Fig. 3g. The model perfectly matched the STEM picture, enabling high-contrast atomic columns and the formation of a large honeycomb closed-packed structure (Fig. 3g (iii)). STEM image simulations predicted that Nb_2_C MXene’s favorable termination atomic positions were located above the hollow sites and were orientated toward Nb atoms. Thus, when positioned on hcp sites of the standard MAX phase, an extra layer of Nb adsorbed atoms (adatoms) with O would emerge on the top surface, with subsequent layers on both sides (Fig. 3g). Palisaitis et al. [26] suggested that these Nb atoms might readily attract oxygen from the environment, resulting in unstable Nb and O clusters, eventually destabilizing the honeycomb M_2_X structures and resulting in oxidative degradation of MXenes. The work gave an atomic-level understanding, with implications for refining etching techniques to decrease adatoms on Nb_2_CT_*x*_ MXene and potentially prolong its ambient air-storage stability.

**Fig. 4:**
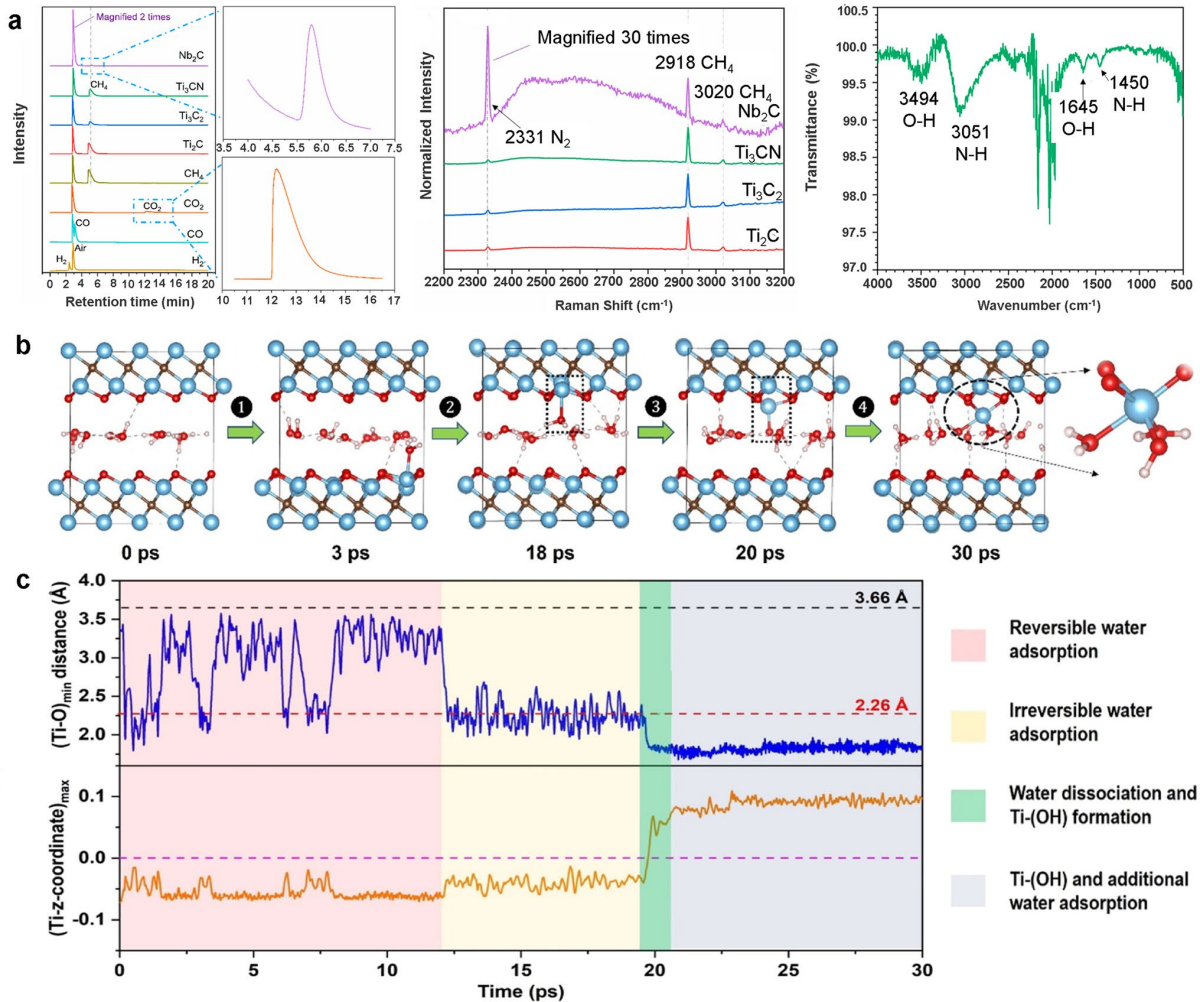
**a** GC analysis of gaseous products produced during oxidation of Ti_2_C, Ti_3_C_2_, Ti_3_CN, and Nb_2_C with zoomed-in peaks for CH_4_ as the main product for Nb_2_C oxidation with CO_2_ reference peak, corresponding Raman spectra of the gas bubbles released during the oxidation of these MXenes in water, and ATR-FTIR spectrum of fully degraded Ti_3_CN dispersion [40]. Copyright 2017, American Chemical Society. **b** Snapshots of water trapped in Ti_3_C_2_O_2_ during a 30 ps FPMD simulation with four phases of water attacking the surface: **(1)** reversible water adsorption on Ti, **(2)** irreversible water adsorption, **(3)** water dissociation and Ti pullout and **(4)** additional reactivity of water molecules as it pulls out Ti, **c** corresponding key distances representation during water attack showing changes in maximum z-coordinate of Ti in relation to surface O layer with dashed lines black and red depict the change in the sum of van der Waals radii and covalent radii of Ti and O in MXene during its interaction with water [41]. Copyright 2022, American Chemical Society

Until recently, oxidation in MXene was assumed to be accomplished equally by water and dissolved oxygen. However, the recently reported work by Huang et al. [40] justified the key role of water in the oxidation of MXenes rather than dissolved O_2_. The gas–solid analysis of different MXenes, such as Ti_2_C, Ti_3_C_2_, Ti_3_CN, and Nb_2_C, during their transformation to oxides in water using gas-chromatography (GC) and Raman spectroscopy, respectively (Fig. 4a) confirmed the generation of CH_4_ from carbide-MXene, and additionally, ammonia in the case of Ti_3_CN proven by the presence of N − H bands of NH_4_^+^ present in its solid residue (Fig. 4a). Since the analysis was carried out in closed vials and the amount of air was constant in each sample, with the assumption of N_2_ inability to react with MXenes, the produced CH_4_ was regarded as the product of direct hydrolysis of MXenes in water. More recently, Wu et al. [41] interpreted the water molecules’ interaction with MXenes using first principles molecular dynamics (FPMD) simulations at room temperature. The simulations were based on tracking the interaction of chemisorbed water molecules within one and two molecular layers of Ti_3_C_2_O_2_ MXenes. The simulation showed the atomic-insight configuration of water-MXene interaction by measuring the proximity of water O atoms, and surface Ti atoms of MXenes Ti_3_C_2_O_2_ denoted as (Ti–O_min_). Accordingly, the Ti–O_min_ bond length fluctuated between 2.0 and 3.5 Å in the first 12 ps, suggesting that the initial adsorption of water on Ti-atoms is reversible, which later evolves into physisorption (3.66 Å) based on the sum of van der Waals radii of Ti and O atoms. It was shown by following the Z-coordinate of the Ti-atoms to the O-atoms of MXene that the adsorption of water slightly pulls the Ti-atoms outwards, resulting in surface reconstruction (Fig. 4b) and making the adsorption irreversible while polarizing the water molecules. This process forms a Ti–OH group near the surface where the much-exposed Ti atoms are thus susceptible to oxidation by attacks from the neighboring water molecules. The hydrolysis of MXene could also be viewed from the perspective of bond formations where initial adsorption of water results in Ti-OH_2_ bonds (2.16 Å) and an additional (O–H) (0.96 Å) bond, which would form a hydrogen bond with neighboring water molecules. As the polarization increases, Ti-OH_2_-OH_2_ distances decrease until the Ti–O-H–H bond is broken, and the H-OH_2_ bond is formed, resulting in an exposed Ti–OH bond, which is then further oxidized by water. The attack of water molecules can also be seen from the perspective of the Ti-C bond, where early water adsorption (reversible) changes the length from 2.25 to 2.75 Å, which eventually reaches 3.0 Å due to chemisorption (irreversible), allowing the Ti-C bonds to loosen and eventually break, allowing the Ti–C bond distance to oscillate near 3.77 Å (Fig. 4c). Interestingly, the work not only predicted that the Ti-atoms are a crucial site for oxidation but that the O atoms of water molecules initiate the initial contact with -O terminated MXenes, suggesting that the oxidation process in MXenes can be significantly influenced by controlling the orientation of water molecules or by surface-functional group alternation. Subsequent simulations in proton-added water and with the MXenes-surface termination set to -OH corroborated these hypotheses, demonstrating that water molecule attack was greatly hindered in both cases.

## Strategies to Mitigate MXenes Oxidation

The oxidation stability of MXenes in their native colloidal form is essential for their widespread applicability. The above discussion shows that the MXenes dispersion in an aqueous solution involves a complex oxidation mechanism with its rate depending on multiple factors that range from MXenes compositional characteristics to its colloidal environment. While some studies have shown that dissolved oxygen plays a role, more recent studies have indicated water’s dominance in the hydrolysis of MXenes. Nevertheless, improving the oxidation stability of colloidal MXenes is gaining immense scientific attention, with new promising strategies reported over time to delay or mitigate oxidation. Here, converting MXenes into solid forms, such as powder or film [42], is one efficient way to extend their shelf-life; however, regaining their colloidal state necessitates an extra step of sonication, which poses a risk of rapid oxidation or flake shattering into smaller sizes. Among the reported strategies, MAX phase alteration [43], deaerated atmospheres [22], organic solvents [44], and antioxidants [45] have also proven effective in delaying the oxidation of MXenes. However, an effective method that could completely halt the spontaneous oxidation of MXenes in an aqueous environment is yet to be established. Taking the example of Ti_3_C_2_T_*x*_, it is well established that controlling the storage environment, such as deaeration (O_2_ removal) and lower temperature, could significantly lower oxidation kinetics [18].

**Fig. 5:**
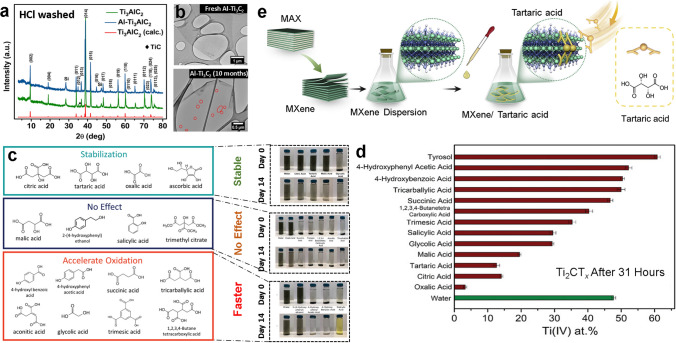
**a** XRD pattern of excessive Al-containing MAX phase (Al-Ti_3_AlC_2_) and typical counterpart Ti_3_AlC_2_ after acid washing, **b** TEM images of high-quality fresh Al-Ti_3_C_2_ MXene and after 10 months of storage [46]. Copyright 2021, American Chemical Society. **c** Various antioxidant molecules and their antioxidation ability and corresponding digital photographs of Ti_3_C_2_T_*x*_ dispersion during 14 days of storage, **d** the variation of Ti (IV) content of MXenes after 31 h of storage in aqueous media containing different antioxidants [45]. Copyright 2022, Wiley–VCH. **e** Schematic illustration depicting the formation of MXene-tartaric acid composite [19]. Copyright 2021, Elsevier

The quality of the MAX phase is particularly important since oxidation begins at defect sites on MXene-flakes, which are partly inherited from the precursor MAX phase. Thus, combining the MILD synthesis approach with a high-quality MAX phase precursor with excellent crystallinity and larger-grain size may produce MXenes with fewer surface defects. For example, colloidal Ti_3_C_2_T_*x*_ produced from MAX phases derived from graphite, carbon lampblack, and TiC as carbon source exhibited distinct differences in colloidal stability and physicochemical features, such as conductivity [43]. In comparison with carbon lampblack (*τ* = 5.1 days) and TiC (*τ* = 4.8 days), MXene synthesized using a graphitic powder-based MAX phase realized greater colloidal stability (*τ* = 10.1 days) and conductivity. This variation in stability resulted from morphological differences in their precursor MAX phase (Ti_3_AlC_2_) imparted by distinctive carbon sources. Thus, properly regulated synthesis parameters might significantly improve MXenes colloidal stability. Mathis et al. [46] also demonstrated the alteration of the MAX phase to prolong the stability of MXenes. Excessive Al in the parent MAX phase (Al-Ti_3_AlC_2_) rendered highly stoichiometric Ti_3_C_2_ layers, which remain stable in aqueous settings for 4 months. Accordingly, sintering a mixture of TiC, Ti, and Al powders with excessive Al results in an intermetallic complex (TiAl_3_) (Fig. 5a), which may be removed by acid-washing. On the other hand, the excess molten metal (Al) increases the MAX phase grain quality and structural ordering, resulting in a highly stoichiometric MAX phase that yields high-quality MXenes. The TEM analysis demonstrated that the high-quality MXenes (Ti_3_C_2_) remained unoxidized for 4 months until the creation of pinholes in the flakes became evident after 10 months (Fig. 5b), suggesting the onset of oxidation.

Storage life improvement has also been achieved through careful management of the dispersion environment, which involves either eliminating oxygen and water from the mixture or drastically reducing the rate at which they interact with MXene-flakes. While transferring Ti_3_C_2_T_*x*_ into organic solvents such as DMSO, DMF, NMP, PC, and ethanol (EtOH) could maintain its shelf-life of 96 h, comparable to newly delaminated MXenes. MXenes with numerous surface functional terminations, such as –OH, = O, and –F, have shown poor stability in solvents such as toluene and hexane [47]. Though the approach is efficient, good dispersibility of pristine Ti_3_C_2_T_*x*_ is limited to only a few polar organic solvents, and transferring MXenes from an organic environment to an application-needed environment (water) is yet another challenge. Surface functional termination alteration has been proposed to improve MXenes organic solvent dispersibility and, thus, storage life. Maleski et al. [47] showed that the functionalization of multilayer exfoliated Ti_3_C_2_T_*x*_ with di(hydrogenated tallow)benzyl methyl ammonium chloride (DHT) could effectively alter MXenes surface characteristics from hydrophilic to organophilic extending their dispersibility to previously difficult solvents like hexane decalin, chloroform, and toluene for at least 10 days.

Since oxidation initiates at the surface-edge defects, surface passivation of these defects by charged species such as polyions offers a better stabilization strategy in its native aqueous form. Natu et al. [48] demonstrated the potency of polyphosphates, polyborates, or polysilicate to edge-passivate Ti_3_C_2_T_*z*_ and V_2_CT_*z*_ colloidal suspensions due to the similarity of their surface chemistry to clays. Accordingly, the storage of Ti_3_C_2_T_*z*_ and V_2_CT_*z*_ in open-air aqueous settings was monitored for a month and 3 weeks, respectively, with morphological and compositional analysis confirming excellent surface passivation with no evidence of TiO_2_ formation. The passivation process followed a simple ionic-interaction mechanism in which negatively charged oxygen functionality in polyanion efficiently interacts with positively charged MXenes edges, thereby capping the sites and preventing their contact with water or dissolved oxygen. More recently, Zhao et al. [49] proposed using L-ascorbate as an anti-oxidant for Ti_3_C_2_T_*x*_, with evidence supporting its stability in the open-air atmosphere. The L-ascorbate-associated MXene maintained its crystalline structure, dispersion quality, conductivity and chemical composition in dried and dispersion form even after 21 days of storage. The reactive molecular dynamics simulation supported the edge-capping mechanism involving the loss of Na^+^ cation from L-sodium ascorbate and the association of the corresponding anions at the edges of the Ti_3_C_2_T_*x*_, preventing its interaction with water molecules. Zhao et al. [45] later expanded the study to systematically examine the interaction of three classes of organic anti-oxidants with Ti_*n*+1_C_*n*_T_*x*_ MXenes. Here, three classes of molecules (-hydroxy acids, polycarboxylic acids and phenolic compounds) were examined for their antioxidation activity for MXenes (Ti_3_C_2_T_*x*_ and Ti_2_CT_*x*_) during a storage period of 14 days. Though the examination of antioxidation efficiency relied solely on XPS measurement of stored MXenes, a relationship was established between antioxidant molecule structure and its ability to inhibit oxidation. Citric acid, tartaric acid, and oxalic acid were particularly efficient in inhibiting the oxidation of Ti_3_C_2_T_*x*_ and Ti_2_CT_*x*_ MXene nanosheets, while a few polycarboxylic acids and phenolic compounds promoted the oxidation (Fig. 5c). The structure-efficiency relationship was developed on the premise that effective antioxidants, such as citric acid or tartaric and oxalic acid-based MXene dispersions had significantly negative zeta potentials and lower hydrodynamic diameters, which confirms a high colloidal dispersibility. Oxalic acid was recognized as an optimal antioxidant among diverse compounds owing to the lower Ti^4+^ content (1.7%) of the corresponding MXene dispersion compared to bare MXenes (9.9%) during the 14 days of storage (Fig. 5d).

Wang et al. [17] demonstrated the use of low-cost inorganic salts such as NaCl, LiCl, and CaCl_2_ to stabilize MXenes in an aqueous system by decreasing the activity of water within MXenes dispersion. The hydration effect of these salts was primarily responsible for the decline in the ratio of free-water molecules and their interaction with MXenes, thereby allowing the aqueous Ti_3_C_2_T_*x*_ to be stable for 400 days with a minimum compromise of their intrinsic properties. Zhang et al. [19] reported a more practical utilization of organic capping agents by employing tartaric acid as a multifunctional antioxidant, successfully suppressing MXenes oxidation and encouraging its composite formation with PEDOT: PSS (Ti_3_C_2_T_*x*_/PEDOT: PSS) (Fig. 5e). Accordingly, a 25 wt% tartaric acid could efficiently limit the oxidation of MXenes in aqueous dispersion even at an elevated temperature of 60 °C, which was unlikely for the untreated MXene. Here, the multifunction of tartaric acid included not only the capping of edge-based Ti-ions and defects but also inducing partial dissociation of PEDOT: PSS complex to replace the insulating PSS with tartaric acid and promote crosslinking between PEDOT nanofibers and MXenes, enhancing the structural stability of the composite in water for more than 2 weeks. The resulting composite (ta-MXene/PEDOT: PSS) demonstrated improved electrical conductivity of 2,240 S cm^−1^ (4-folds) compared to untreated MXenes (552 Scm^−1^) with no physicochemical changes.

At present, the addition of anti-oxidants to stabilize MXene is a relatively easy and efficient route to prolong the shelf-life storage of MXenes in their aqueous form [17]. However, the overall stabilization efficiency depends on the nature of the anti-oxidants (structure) and their mode of interaction with MXenes [45]. Though effective, the use of anti-oxidants presents a concern in terms of quantity since their content is significantly greater than MXenes concentration. Moreover, removing these anti-oxidants from the colloidal dispersion is also a challenge before using these MXenes for diverse applications. For example, when utilized in a biosensor, the inclusion of organic molecules as capping agents raises the possibility of their interaction with a surface-bound biological entity. Nonetheless, the science of antioxidation strategies for MXenes is new, requiring in-depth investigation of molecule-MXene interaction and their atomic level chemistries to fully understand the edge or face-specific capping of MXenes [50]. In addition, the antioxidation strategies should also be extended beyond Ti_3_C_2_T_*x*_-MXene members to understand the distinctive molecular-MXene interactions and develop a universal mechanism applicable to all forms of MXenes.

Among unconventional routes, carbon coatings [51] and ionic liquids [52] have also been explored for improving the oxidation stability of MXenes. Moreover, the grafting of MXenes has also been explored to improve their storage stability and dispersibility in organic media. Lim et al. [53] explored the possibility of grafting lipophilic octyltriethoxysilanes (OTS) on Ti_3_C_2_T_*x*_ to improve stability and dispersibility in hexane. MXene grafted with water-insoluble OTS was highly hydrophobic with a water-resistivity of 30 days and an interfacial tension (IFT) of 46.69 ± 0.04 mN m^−1^ for the corresponding films immersed in hexane. Similarly, Bian et al. [54] allowed a room-temperature reaction between octadecyl isocyanate and Ti_3_C_2_T_*x*_ MXene to introduce long-chain alkanes on MXene, extending its aqueous aging life to 3 weeks. Despite longer shelf-life, these hydrophobic MXenes had limited applicability for aqueous system applications. Thus, the aqueous colloidal stability of Ti_3_C_2_T_*x*_ flakes was improved by grafting with zwitterionic polyelectrolytes (PE), which improved the aqueous storage of MXenes for up to 60 days [55]. In the case of grafting, the most relevant research is focused on visual dispersion/film quality evaluation to determine their stability in air or water. This visual reliability might be mistaken for oxidative stability (oxidation inhibition). Thus, advanced methods like in situ XRD or Raman may be adapted to offer a clear insight into the grafted films’ oxidation status.

Understanding MXene’s oxidation as a function of temperature change is essential for the development of low-temperature stabilizing strategies. Since the oxidation of MXenes is primarily caused by the presence of water, the dehydration of MXenes at high temperatures and/or in different atmospheres might give crucial information regarding the role of entrapped water molecules and their interaction inside MXenes. For example, Thakur et al. [56], based on in situ Raman analysis, provided insights into the oxidation of V_2_CT_*x*_ that occurred at bulk and surface with high temperatures. Thakur et al. [56] postulated that V_2_CT_*x*_ in different atmospheres (N_2_, CO_2_, air, and H_2_) generally behaves differently, mainly due to the different content of trapped water molecules within the bulk structures. In N_2_, surface oxidation was seen up to 600 °C, but in CO_2_, total oxidation was observed at 300 °C. Unlike air, where V_2_CT_*x*_ completely oxidizes at 400 °C, the H_2_ environment reduces surface-generated VO_*x*_ species and terminations above 300 °C. The in situ Raman-based correlation between the release of water molecules and structural and chemical change in V_2_CT_*x*_ suggested partial oxidation occurred via dehydration which lowered the layer-to-layer distances at 400 °C, and total dehydration and oxidation occurred above 700 °C. The study speculated water confinement within MXenes layers and its release in packets when heated. Thus, the confined water’s interaction would differ in bulk and at the surface, requiring step-wise temperature changes to dehydrate MXenes. The study paved a new route toward understanding water–solid interaction in MXenes and defining the optimum dehydration process for enhanced stability. Since the dehydration temperature could vary with the water-MXene intra-layer interactions, the location of these trapped water molecules and understanding their interactional chemistry is crucial and still open to investigation.

The influence of surface-termination variation on Ti_3_C_2_-MXene film stability under high temperature (70 °C) and harsh humid (100%) conditions were later investigated by Lee et al. [42], where hydrogen annealing allowed the reduction of MXenes surface hydroxyl groups to oxygen terminations. Here the change in electrical resistivity was used as a measure of oxidation of the film, and the presence of TiO_2_ was verified by Raman and XPS spectroscopy. The reduced hydroxyl groups and short-term sintering (100–900 °C for 30 min) to remove adsorbed water were suggested to improve the film’s oxidation stability. Surprisingly, hydrogen post-annealing regained the initial-sheet resistance, which, when investigated using XPS, revealed the existence of tiny TiO_2_ nanoparticles (5 nm), indicating the method’s failure to halt MXenes oxidation.

Heat treatment at different temperatures has also been explored to alter the MXene surface features and passivate the surface or edge defects (oxidation-prone sites) on MXenes. For example, Zhao et al. [57] proved that a layer of TiO_2_ formed post-annealing of MXenes film in an inert Ar atmosphere can act as a protective barrier and prevent water penetration in the structure. The compact sandwich-like MXene/TiO_2_ arrangement prevented Ti_3_C_2_T_*x*_ MXenes aggregation and preserved the internal structural configuration allowing the annealed films (600 °C) to remain stable in water for over 10 months. Although promising for edge-passivation of high-quality MXene-film, controlling the TiO_2_ content is still a challenge, in addition to understanding the interactions between the surface-TiO_2_ and bulk of MXenes. Zhang et al. [58] advanced the low-temperature stabilization of MXenes to more applicable freeze preservation. In this case, storage of Ti_3_C_2_T_*x*_ below -20 °C could prolong its storage period to 650 days without any changes to its physical characteristics, e.g., color. While this is feasible at the laboratory scale, freezing MXenes on a large scale would not be commercially practical.

In the case of Ti_2_CT_*x*_ MXenes, thermal stability is generalized below 250 ºC in inert atmospheres like N_2_ and Ar. An exception was reported by Lai et al. [59] in which Ti_2_CO_*x*_ remained stable even at 1,100 ºC, which is unlikely as the phase stability of Ti_2_C is achieved at much lower temperatures. Although considerable efforts have been made to comprehend the oxidation of MXenes at various temperatures and environments, we believe that expanding the high-temperature stability research beyond the Ti_3_C_2_T_*x*_ member and incorporating phase-diagrams in combination with detailed Raman analysis could provide a clearer picture of MXenes phase-transformation, which could influence its applications involving heat-treatment processing.

## Challenges and Future Prospects

The oxidation of MXenes is a bottleneck challenge requiring immediate attention before MXenes, and their derivatives may be progressed for various applications. Compared to MXenes in free-standing film form, the oxidation rate is much higher in their aqueous dispersion form, whereas the precise mechanism by which MXenes oxidize in their aqueous state is still under debate. In the case of Ti_3_C_2_T_*x*_-MXenes, an early investigation revealed that oxygen and water have a complex role in the onset of oxidation, which was eventually narrowed down to water as the primary culprit for Ti_3_C_2_T_*x*_ hydrolysis. Several studies have explored the oxidation of MXenes, notably Ti_3_C_2_T_*x*_, in various storage conditions and identified different parameters influencing the oxidation rate, ranging from the pH of the dispersion to the nature, size, and concentration of MXenes.

Oxidation has also been considered from a materials perspective, with MXenes edges, defects, and vacancies identified as oxidation-prone locations. Thus, eliminating surface defects that may have been inherited from the MAX phase or generated during mechanical and chemical exfoliation may impact the oxidation susceptibility of MXenes. Nevertheless, a comprehensive understanding of how water molecules interact with these sensitive sites remains elusive. Initial studies emphasized the formation of internal fields near these defect sites, which facilitates the transportation of Ti-ions from bulk to the edges, exposing them to water or oxygen for TiO_2_ nucleation, which later grows, resulting in voids in MXenes, leading to their deterioration. In contrast, FPMD simulations-based understanding of water-MXene interaction indicates the adsorption of water molecules on MXenes, which then slightly pulls the Ti-atoms outwards, resulting in MXenes-surface reconstruction and formation of Ti–OH group near the surface, which ultimately leads to hydrolysis of MXenes. Since the defect sites in MXenes are critical to its oxidation, precise control of pre-synthesis conditions such as MAX phase quality and synthesis conditions such as MILD methods could significantly delay the oxidation process in the MXenes. Here, emphasis should be given to scalable, environmentally friendly synthesis processes that could result in high-quality MXenes with low surface defects. Moreover, the MXenes synthesis methods usually produce moderately different compositional characteristics, i.e., surface termination. Thus, a deep insight is required to control or design a specific crystal structure with desired functionalities and fewer atomic defects using optimized synthesis protocols to ensure maximum stability. The present understanding of these characteristics often relies on the empirical view rather than a solid theoretical mechanism. For example, the complexities in the chemical etching process of Ti_3_C_2_T_*x*_ make it difficult to accurately predict the concentration of atomic defects and surface termination using simple thermodynamic and kinetic modeling. More importantly, the atomic defect and pores’ formation mechanism are still unclear.

To comprehend the oxidation process, especially beyond Ti_3_C_2_T_*x*_, a deeper examination is required to understand the mechanism on an atomic level. Currently, the storage period of Ti_3_C_2_T_*x*_ is variable, resulting in a debatable interpretation of its oxidation process. Several competitive reactions, i.e., adatoms, trapped H_2_O and edge-oxidation, are coherently contributing to the oxidation process. Thus, a solid background is needed to develop a relationship between the surface or edge-related chemistry to the bulk of MXenes and their real-time behavior under various environmental settings, i.e., temperature and atmosphere. Standard methods, such as color and optical signature, are often used to examine the MXenes dispersion stability (oxidation occurrence). However, the resulting interpretation may not accurately reflect the true MXenes oxidation state. Thus, emphasis should be given to develop new methods to precisely identify and quantify oxidation, which requires more dependence on in situ techniques for an accurate atomic-level status of MXenes. Moreover, the role of water molecules, and their interaction within bulk and surface, besides surface termination interaction, also requires investigation for a clear understanding of the hydration layer formation over MXenes. The thermal stability of MXenes is presently confined to a few MXenes-members. We believe phase diagrams of nonstoichiometric transition metal carbides to model/predict certain phase stabilities will help estimate MXenes thermal transformations.

The antioxidation strategies that offer simple and efficient routes to stabilize MXenes in their natural aqueous dispersion form are presently limited to anticipating an improvement in MXenes’ shelf-life. There is still more work to be done on determining the overall influence of these anti-oxidants on MXenes performance in various applications. As the antioxidation strategies largely rely on the surface capping of MXenes, more systematic research is needed to fully understand the molecular-MXene interactions and the stereospecific orientation of the antioxidant over the MXenes surface. The concentration of these antioxidants and their complete removal is also a concern. Thus, research direction should be diverted to exploring more potent antioxidant molecules that could effectively stabilize MXenes at lower doses with ease of removal or without the need to remove them.

In the last decade, an exponential expansion has been seen in MXene materials research, leading to the emergence of innovation in nanotechnology and materials engineering. The increasing compositional variety and technological superiority demonstrate MXenes’ infinite potential for future expansion. Here, improved synthesis processes, MAX phase quality, and efficient stabilization methods that can provide regulated surface moieties, optimum atomic defects, and flake size are all possible avenues to improve MXenes’ intrinsic stability. Interestingly, where oxidation significantly impacts the physicochemical characteristics of MXenes, their transformation into oxides has also inspired in situ strategies where controlled oxidation has facilitated the fabrication of MXene/metal oxide heterostructures with prospective uses in photocatalysis and energy storage materials. It is therefore important to encourage the scientific community to investigate MXene oxidation not only as a challenge but also as an opportunity to broaden the scope of MXene and its derivatives materials for diverse applications.
